# Persistent weight loss with a non-invasive novel medical device to change eating behaviour in obese individuals with high-risk cardiovascular risk profile

**DOI:** 10.1371/journal.pone.0174528

**Published:** 2017-04-12

**Authors:** Peter von Seck, F. Martin Sander, Leon Lanzendorf, Sabine von Seck, André Schmidt-Lucke, Mirja Zielonka, Caroline Schmidt-Lucke

**Affiliations:** 1 Medical Practice, Wiesbaden, Germany; 2 Dental Clinic, Frankfurt, Germany; 3 Department of Health Care Management, Technical University Berlin, Berlin, Germany; 4 MSD SHARP & DOHME GMBH, Berlin, Germany; 5 Department of Internal Medicine, Hygiea Hospital and Medical Practice, Berlin, Germany; 6 Charité University Berlin, Berlin, Germany; 7 Medico-academic Consultings, Berlin, Germany; Vanderbilt University, UNITED STATES

## Abstract

In evidence-based weight-loss programs weight regain is common after an initial weight reduction. Eating slowly significantly lowers meal energy intake and hunger ratings. Despite this knowledge, obese individuals do not implement this behaviour. We, thus tested the hypothesis of changing eating behaviour with an intra-oral medical device leading to constant weight reduction in overweight and obesity.

Six obese patients (6 men, age 56 ± 14, BMI 29 ± 2 kg / m^2^) with increased CVRF profile were included in this prospective study. All patients had been treated for obesity during the last 10 years in a single centre and had at least 3 frustrate evidence-based diets. Patients received a novel non-invasive intra-oral medical device to slow eating time. Further advice included not to count calories, to avoid any other form of diet, to take their time with their meals, and to eat whatever they liked.

This device was used only during meals for the first 4 to 8 weeks for a total of 88 [20–160] hours. Follow-up period was 23 [15–38] months. During this period, patients lost 11% [5–20%] (p<0.001) of their initial weight. At 12 months, all patients had lost >5%, and 67% (4/6) achieved a >10% bodyweight loss. In the course of the study, altered eating patterns were observed. There were no complications with the medical device. Of note, all patients continued to lose weight after the initial intervention period (p<0.001) and none of them had weight regain.

With this medical device, overweight and obese patients with a history of previously frustrating attempts to lose weight achieved a significant and sustained weight loss over two years. These results warrant the ongoing prospective randomised controlled trial to prove concept and mechanism of action.

**Trial registration:** German Clinical Trials Register DRKS00011357

## Introduction

Substantial and sustained increase of total energy intake worldwide has led to increased body weight across the global population, with an estimated 1.5 billion overweight individuals [1]. Obesity is a significant public health problem [2] [3], resulting in a heightened risk of diabetes, cardiovascular disease, cancer and other illnesses [4] [5] [6] [7]. The abundance and permanent availability of high-caloric food and the habitual sedentary lifestyle on the background of evolutionary engraved and perinatally imprinted physiological response patterns are key factors for individual and global weight gain.

Consequently, rectifying this imbalance by caloric restriction and increased physical activity form the basis in the management of evidence-based interventions of obesity [8] [9]. However, although programs including dietary restrictions, physical activity and behaviour modification can and do lead to weight loss [10], over the long term, the majority of individuals regain the weight they have lost [11]. Weight stability after weight reduction programs requires profound and permanent changes in lifestyle and eating behaviour [12]. Not only is weight regain frustrating for the individual, weight fluctuation is also an independent predictor for disease burden [13]. Despite the prevalence of evidence-based diets and programs, losing weight and preventing weight regain in overweight and obesity remains a serious challenge. It is apparent that the effects of diet and exercise interventions alone are not sufficient to support long-term maintenance of a reduced weight [14].

Obesity can be regarded as a neurobiological disease with a psychological element. The mechanisms underlying flawed eating behaviour are complex. Several physiologic adaptations after voluntary weight loss favour weight regain. These involve appetite-related hormones [15], the complex neuro-hormonal system, hedonic and reward circuits, and changes in energy expenditure. [12] [16]. The restraint theory provides an attempt to synthesise the behavioural, cognitive and affective components of the food intake process [17]. Unusual eating patterns are proposed to develop as a result of the stress associated with the demands of continual self-control [18], with food intake determined by a balance between the desire to eat and the aspiration to diet. [19]. That is, cognitive processes override physiological hunger and satiety cues [20]. Over the long-term, the more powerful emotions may override cognitive control and result in over-eating, which is often seen in individuals after consequent dieting.

With the acceleration of obesity rates and few effective obesity treatment options, new strategies addressing changes of eating behaviour are urgently needed. Successfully maintaining weight loss requires a permanent change of behaviour.

We thus tested that hypothesis that a relatively short period of using an intraoral device to change eating behaviour in overweight and obesity could lead to a constant and long-lasting weight reduction.

## Materials and methods

This was a prospective investigator-initiated, open-label therapy trial to test an innovative oral medical device designed to reduce chewing area (patent application DE102012015839A1, EP000002695588A1, US-Application no. 13/961,024). The study was conducted in a single centre in a specialist practice from July 2012 to January 2015. Recruitment of patients was from July 2012 to August 2014 Patients with clinical indication for weight reduction were prospectively recruited to test the intraoral device. Sponsor of this study were the physician and patent holder and the collaborating orthodontist. The physician conducted the study together with assisting personnel. He took no part in analysis or preparation of the manuscript. All study participants gave written informed consent. This study in accordance with the Helsinki convention and was approved by the locals Ethics Committee (FF 1/2015) Landesärztekammer Hessen (Frankfurt, Germany). The study has been registered in the German Clinical Trials Register on 8.12.2016 and has been assigned the number DRKS00011357. The study was initiated and carried out by a senior general practitioner and his befriended patients to prove the concept. They were not aware of the requirement to register.

### Participant selection

Participants were recruited in the practice if they fulfilled the following eligibility criteria: BMI 25.0–29.9 kg/m^2^ with other risk factors (i.e. hypertension, diabetes, abdominal obesity) or BMI over 30.0 kg/m^2^; at least 2 previous evidence-based weight management programmes with consecutive weight regain in the last years (≥ 1kg / year); will to reduce weight; age 18–75 years; increased cardiovascular risk profile to develop cardiovascular disease by considering age >65 years, male sex, hypertension, diabetes, and smoking for coronary artery disease as single cardiovascular risk factors (more than 3 individual factors [21]); dental examination within the past 12 months; good oral health and functioning occlusion. Exclusion criteria were participation in a weight reduction programme during the last 6 months prior to this study, weight fluctuation of ≥ 5% over the last year, uncontrolled eating behaviour, clinical or biochemical evidence for the presence of concomitant chronic inflammatory disease, presence of any other significant uncontrolled medical condition or any other treatment known to alter appetite or weight, obesity due to secondary disorders, inability to understand the consent form, participation in or consent to participate in another study, malignant disease, wearing dental prosthesis, less than 10 teeth /jaw with maximally mild to moderate periodontal disease, and poor oral/dental health (as confirmed by the trial dentist).

### Study protocol

**Fig 1:**
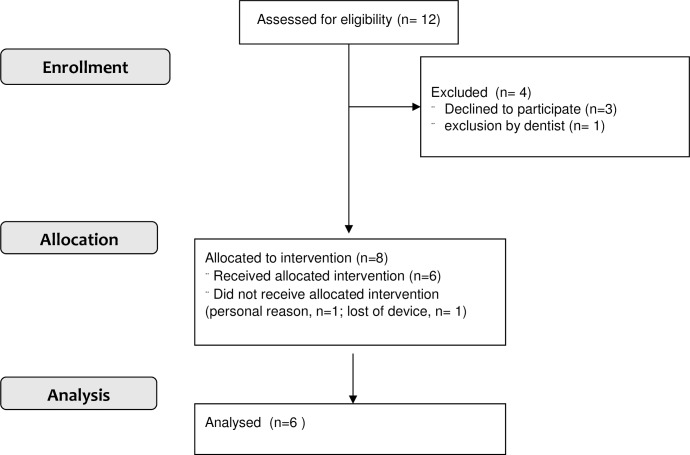
Flowchart.

Patients who fulfilled the above-mentioned inclusion and exclusion criteria were sent to the orthodontist (consultant to the project) for baseline testing and preparation of dental impressions using a purpose-designed kit (Fig 1). The device was custom-made for each individual from the individual’s dental impressions. When needed, the orthodontist adjusted the tool to ensure that it fit securely prior to commencement of use. Patients were advised to refrain from using the device if they felt any problems and to consult the orthodontic at their earliest convenience. On the day of device dispensation (day 1), each participant attended the practitioner for a consultation and baseline measurements. Participants were encouraged to use the device for all meals. Further advices included to strictly avoid sweetened or sugary drinks, avoid any cognitive control over their eating behaviour and to eat what they felt was best for them: in other words, to refrain from any conventional caloric-restricted diet or meal supplements, to eat when and what they liked, to take their times with their meals, savouring every bite and eating mindfully [22], and to take up physical activity only if they felt like it. Patients were advised to use the device for all meals for the first 8 to 12 weeks (Phase I). They were told not to use the device thereafter (Phase II), but allowed to place the box with the device next to them when eating for remembrance.

Participants attended follow-up appointments in the practice at weeks 2, 4, 6, 12, and at months 6, 9, 12, 15, 18, 24, 30 and 36. Weight, compliance, adverse events (AEs), and qualitative feedback were obtained during each consultation. For AEs, a standardised questioning at each contact was performed.

### The intraoral device

The novel intraoral device has been designed to slow the eating process, with the intent of prolonging the chewing process and delaying the swallowing of a single bite in order to improve the function of physiological satiation mechanisms. Additionally, the duration of the single bite in the mouth should be extended to intensify the tasting of the food. Thereby the device aims to promote mindful eating and a long-lasting change in eating behaviour.

**Fig 2:**
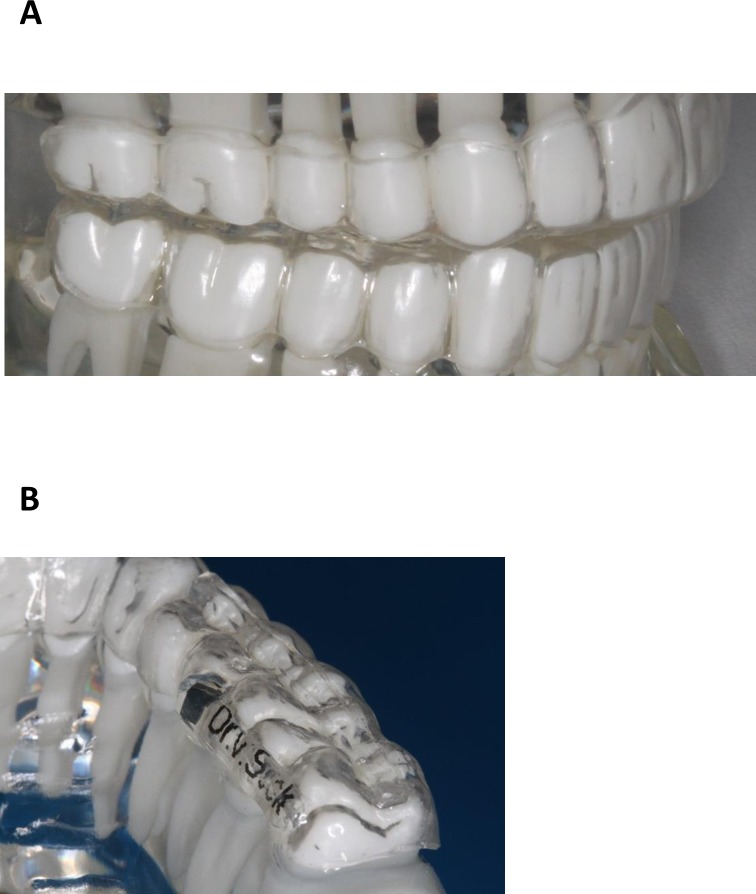
Medical device on dental model. (A) Splints are almost invisible on the front teeth of the upper and lower jaws. (B) linear increase over the upper posterior molars reduces the chewing area to about one fifth.

The design of the intraoral device consists of two thermoplastic splints made of Polyethylenterephthalat-Glycol (PET-G) for the upper and lower jaw **(**Fig 2). On top of each splint a smooth layer made of Polymethylmetacrylate (PMMA) is formed, which reduces the occlusal surface in centric jaw occlusion. Additionally, a linear increase with a width of about 3mm over the upper premolars and molars further reduces the chewing area. Thus, the occlusal surface is reduced to about one fourth of its original surface. The completely smooth surface of the splint that covers the lower jaw further restricts the chewing process by inhibiting the interlocking of dental cusps. The device is inserted immediately before ingestion of food and is removed afterwards.

### Clinical assessment and questionnaires

Weight was measured at baseline and each follow-up (see above), using the same digital scales. Patients’ historical weight was retrieved from the individual records. At baseline and last follow-up, height, weight and waist circumference were quantified and the body mass index (BMI, kg / m^2^) calculated. Supine blood pressure was measured in the left arm. Venous blood samples were taken at baseline and after 3 and 6 months, as clinically indicated, to assess plasma glucose, insulin, haemoglobin A1c (HbA1c), total, high-density lipoprotein and low-density lipoprotein cholesterol and triglycerides. These samples were taken after an overnight fast. Concomitant medication was assessed at baseline and changes monitored throughout the study.

To exclude participants with uncontrolled eating behaviour, volunteers were screened for uncontrolled eating behaviour using the German version of the Three Factor Eating Questionnaire (TFEQ) [23]. A questionnaire, specifically designed for this study, assessed participants’ acceptability of the intraoral device, eating rates, food choices, comfort, appetite, and meal size throughout Phase I. Responses were noted by the practitioner and evaluated at the completion of the study.

### Compliance

Compliance throughout Phase I of the study period was defined as reported by participants. Good compliance as use of a minimum use of 3 meals/day, 6 days/week; moderate compliance as use of 15 to 18 meals/week; poor compliance as use of 9 to 14 meals/week; less than 9 meals/week was defined as no compliance. These values were averaged to obtain a “mean uses per week” across the Phase I of the study for each participant. Additionally, length of use in weeks and cumulative duration of use in hours were recorded.

### Adverse events and qualitative data

At each visit, participants were asked to report any adverse events; these were recorded, and where necessary, follow-up action was taken by the medical officer or dentist.

### Assessment of eating with the device

In their first week, the last three participants included in the observation volunteered to participate in an additional acute eating experiment with the device. For this, they were asked to eat an identical ad-libidum meal (lunch) on two following days at home without the device (baseline) and on the following day with the device. All volunteers were instructed to eat a light breakfast on the test days. They all reported to have followed the instructions.

Appetites were measured with a German translation of the Visual Analog Scale (VAS) [24] and additional standardised questions assessing chews per bite, duration (in seconds) to swallow, percentage of consumed meal, satisfaction from the meal, awareness of food composition and amount of food consumed, duration of the meal, intake of a snack after 2–3 hours and type of snack following each meal. Changes of eating behaviour and appetite following the meals were assessed by comparison of the individual questionnaires from day 2 with baseline. Food intake motivation after the respective test meals was assessed with the use of a paper version of the VAS, completed directly afterwards on a 100-mm line. The scales assessed: hunger (How hungry do you feel at this moment?), desire to eat (How strong is your desire to eat at this moment?), fullness (How full does your stomach feel at this moment?), and motivation to eat (How much food do you think you could eat at this moment?). These VAS ratings have been determined to be valid and reliable indicators of hunger and satiety VAS. [24]

### Statistical analysis

Data are expressed as mean ± standard deviation (SD). All continuous variables were assumed not to be normally distributed due to the small number of patients included and compared with the Mann-Whitney U test. Comparison of categorical variables was generated by the Pearson χ^2^ test and the Fisher’s exact test. Primary outcomes (weight loss in Phase I and Phase II) were assessed on an intention-to-treat basis. A total of 6 patients will enter this study. The probability is 80 percent that the study will detect a relationship between the independent and the dependent variables at a one-sided 0.05 significance level, if the true change in the dependent variables is 2.484 units per one standard deviation change in the independent variable in a test to find an association if the dependent variable is affected by the treatment. This is based on the assumption that the standard deviation of the dependent variable is 2.

Statistical significance was assumed if a null hypothesis could be rejected at p≤0.05. All statistical analysis was performed with SPSS 21.0.

## Results

### Patient characteristics

**Fig 3:**
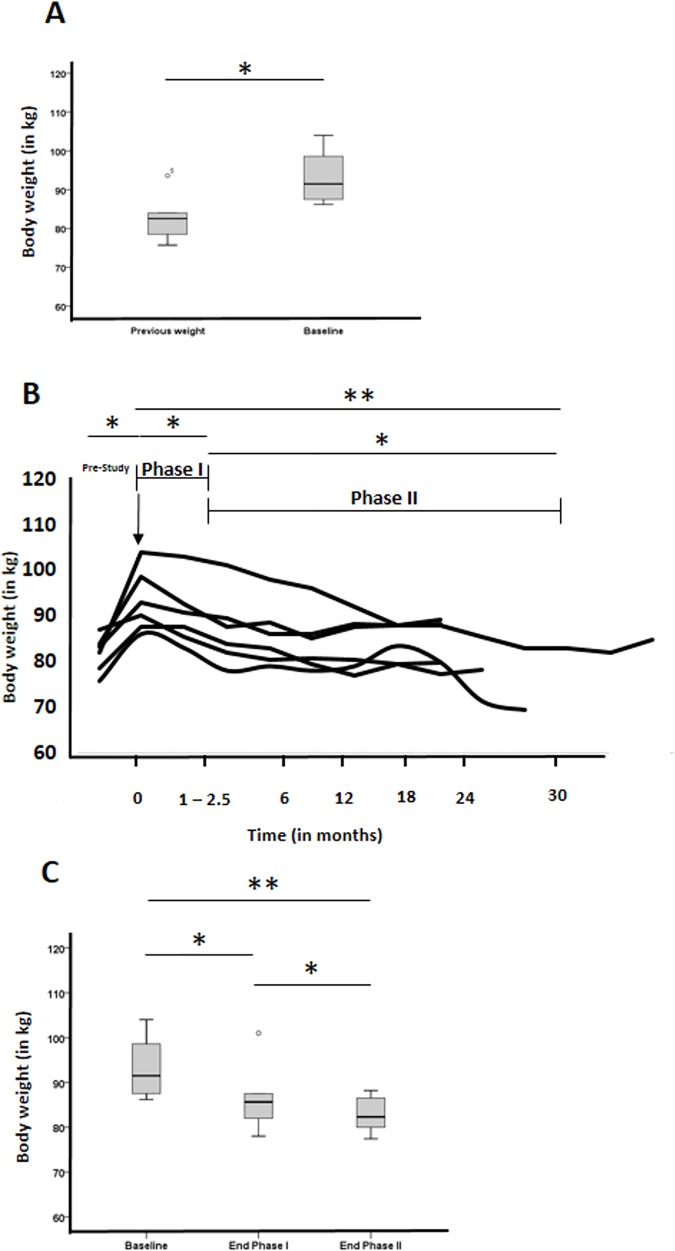
Changes of body weight of participants during the trial. (A) Change of body weight of all participants prior (4 to 12 years) to testing the device and at inclusion (baseline). (B) Individual change of body weight for all participants during the trial prior to the study (pre-study), -> indicated first usage of the medical device, Phase I is the time period where patients used the device (4 to 10 weeks), Phase II is the follow-up period, during which the device was no longer used. (C) Average change of body weight for all participants during the trial. Boxplots’ bar are medians, grey area represent 25 to 75 IQR, and whiskers are 5^th^ to 95^th^ percentiles. * indicates p <0.05, ** indicates p < 0.005.

From among the practice, 12 patients were identified as meeting eligibility criteria and offered to test the novel medical device. Of these, 9 consented to participate. Of these, 1 patient had dental exclusion, 1 lost his device on a longer trip abroad (week 2) and 1 patient felt uncomfortable with the device (week 1). Six patients participated in and completed the study and were used for analysis (Fig 1). None of the patients had a depression or eating pathology on food intake and appetite. The demographic, anthropometric, clinical, and biochemical characteristics of the 6 patients at baseline and at last follow-up are summarised in Table 1. As shown in Fig 3A, patients had a documented average increase of their bodyweight over the last 4 to 12 (8 ± 2.6) years of 10.4 ± 8.3 kg, p <0.05) prior to receiving the device, despite undertaking at least 2 lifestyle modifying programs to lose weight. Follow-up of the study was 15 to 38 (23.2 ± 10.0) months.

**Table 1:** Baseline characteristics and demographic variables.

Parameter	Pre-Study ^1^	Baseline	Last Follow-up	Change	Significance
Age		56 ± 14			
Male gender		100%			
Bodyweight (kg)	83 ± 6	93 ± 7	83 ± 4	^2^ 10.4 ± 8.3 kg	0.01
				^3^ ^-^ 10.5 ± 4.2%	
BMI (kg/m^2^)	26 ± 1	29 ± 2	26 ± 2		0.01
Waist circumference (cm)	n. d.	108 ± 3	98 ± 4	^3^ - 10 ± 4	0.05
SBP (mmHg)		137 ± 8			
DBP (mmHg)		82 ± 4			
HbA1c (%)		6.1 ± 0.8	5.8 ± 0.6		n.a.
n = 2					
Physical activity (h / week)		1.6 ± 0.9	1.7 ± 1.0		
Cumulative number of CVRF		3.8 ± 0.8			

Table legend: CVRF, cardiovascular risk factors; DBP, diastolic blood pressure; SBP, systolic blood pressure

^1^ historical data from patients records (period ranged from 4 to 12 (8 ± 2.5) years

^2^ difference pre-study to baseline.

^3^ difference baseline to last follow-up.

### Weight loss and compliance

All patients significantly lost weight during the initial Phase I (6.8 ± 3.3 kg or 7.3 ± 3.6% of baseline bodyweight after 3 months, p < 0.05, Fig 3B and 3C) where they wore the device. Interestingly, all patients continued to lose weight in Phase II (4.0 ± 4.1 kg during Phase II, p < 0.05) without usage of the device, achieving our primary weight-loss objective of a long-term change of eating behaviour (10.5 ± 4.2 kg or 11.0 ± 4.9% of baseline bodyweight for last follow-up, p < 0.01, Fig 3B and 3C). All patients lost >5% initial bodyweight, and 67% (4/6) achieved a >10% bodyweight loss over the whole study period. When restricting the observation period to 12 months similar success rates were measured, since all patients had lost >5%, and 67% (4/6) achieved a >10% bodyweight loss. Waist circumferences also decreased significantly (−9.4 ± 3.6 cm, p < 0.05).

After receiving the device, patients used the device between 4 to 10 (7 ± 2) weeks. The wide range is explained by a self-reported significant weight loss due to a change in eating behaviour in 2 patients, who were than allowed to eat without the device. Four patients were defined as fully compliant, 1 as moderately and 1 as poor compliant according to the practitioners assisting personnel (2 independent judgements). Cumulative use of the device for the individual patient was 20 to 160 (100 ± 57) hours.

### Safety and acceptability

There were no adverse events with the device reported. All patients reported uncomfortable foreign body sensation with the device that subsided after 1 week (n = 2), 2 weeks (n = 3), or after 4 weeks (n = 1). Further discomfort was reported by 2 patients, related to mild gingivitis that required re-adjustment by the orthodontist (n = 1), intermediate difficulties with biting and chewing some food (salads and certain meat) and attracting another person’s attention (n = 1) during eating and were reported to be “rare” or “very rare”. Apart from these events related to the device, no other incidences occurred. In general, patients reported that handling of the device for insertion and removal was “very good” or “good” in 4 cases and to be “acceptable” in 2 cases. Three patients would recommend the device “unreservedly” and 3 “more yes” (on a scale to five).

### Change in eating behaviour

As shown in Table 2, all patients noticed a change of eating behaviour. Three patients reported using the device as a reminder on rare occasions, whereas the others refrained from using the device as reminder.

**Table 2:** Changes of subjective eating behaviour and quality of life during long-term follow-up.

Description	Value (on a scale of five ordinal values, 1 = best, 5 = worst, unless otherwise indicated)				
	1	2	3	4	5
Reduction of dose and amount of concomitant medication	2	1	3	0	0
Eating with pleasure (1 = more pleasure)	1	3	2	0	0
Prolonged time to eat meals	4	2	0	0	0
Choice of food more “natural”	2	3	1	0	0
Frequency of snacks in between meals (1 = less)	3	2	1	0	0
Counting calories (1 = never)	3	1	2	0	0
Noticed change of behaviour	4	2	0	0	0
Hunger / appetite between meals	4	2	0	0	0
Refreshing sleep	4	0	1	0	0
Increased gut feeling / less cognitive control	4	2	0	0	0
Less tired after meals	6	0	0	0	0
Improved overall health	3	3	0	0	0

Comparison after last follow-up with baseline, Numbers indicate sum of patients’ choice

Changes in food intake were considerable and similar in all three volunteers between eating without (baseline) or with the device, as shown in Table 3. Using the device, there was an increase in chews per bite (3-fold), time to swallow a bite (3.5-fold), and duration of the meal to satiety (3.5-fold), whereas the size of the meal consumed was reduced to 59%. Awareness of composition and amount of food consumed was higher with the device in the acute test meal setting and patients reported during a long-term follow-up that this effect continued.

**Table 3:** Changes of eating and hunger at baseline without (w/o) or with (w) the medical device eating a standardised meal.

Description	Patient						Average Baseline	Average Device	Change (in %)
	1		2		3				
	w/o	w	w/o	w	w/o	w			
Chews / bite	9	25	10	20	7	25	9 ± 1	27 ± 7	300
Time to swallowing (in seconds)	7	20	5	10	5	18	6 ± 2	22 ± 17	366
Percentage of consumed meal (in %)	100	50	100	66	100	50	100 ± 0	59 ± 9	- 41
Duration of the meal (in minutes)	12	25	30	45	10	25	17 ± 11	36 ± 10	212
Awareness of food composition (1 = full, 5 = poor)	3	1.5	1.5	1.5	2	2	2 ± 1	1.8 ± 1	
Awareness of amount of food (1 = full, 5 = poor)	5	2	2	2	4.5	1.5	3.8 ± 1	2 ± 1	
Pleasure from food (1 = full, 5 = poor)	1.5	1.5	3	3	1.5	1.5	2 ± 1	2 ± 1	
Feeling hungry immediately after the meal (1 = no, 5 = very much)	0	0	0	0	0	0	0 ± 0	0 ± 0	
Feeling hungry 2 to 3 hours after the meal (0 = no, 5 = very much)	1	0	1	0	0	0	0.7 ± 0.6	0 ± 0	

## Discussion

The findings of this study support the hypothesis that this medical device is the first non-invasive medical device to reduce weight in overweight and obesity effectively and enduringly. Our data indicate that this weight loss is a result of a sustainable change in eating behaviour. What’s more, the approach of this therapy strongly contrasts with contemporary weight loss strategies in that all patients were instructed to eat whatever they liked.

Treatment of obesity includes food consumption because this is the primary source of caloric intake and manipulating eating behaviour thus is a primary target. Commonly, weight loss strategies to fight individual and populations overweight and obesity include behaviour modification, pharmacotherapy, meal replacements, and more drastically, gastric bypass surgery, or gastric balloon. Despite proven short- and medium-term weight reduction of these evidence-based programs, rapid weight gain is a serious health problem [13] for the majority of patients trying to lose weight.

All patients with increased cardiovascular risk profile who were included in our study had increases in weight despite previous evidence-based strategies to reduce weight over the last years. Participants all lost weight in the first phase, when they used the device for their meals and reported to have changed both the size of their meals as well as the choice of food consumed during this first acute phase of only a few weeks. To verify this finding, an acute experiment was conducted in a subgroup of patients, where patients showed prolonged eating duration, increased awareness of food composition and feelings of satiety, although they had eaten smaller meals when eating with the device. Satisfaction and compliance with the medical device in our study was acceptable after initial discomfort and no adverse events occurred.

Thus, this device is different from other medical intra-oral devices that attempt to prevent an individual from eating but do not change eating behaviour.

There is a strict relationship between bite size and food intake [25] as well as oral health status and involuntary weight loss [26]. First experiences have been made with intraoral medical devices to restrict food consumption in obesity. An oral volume restriction device modified the subjects’ bite size, but this was not associated with changes in food consumption, hunger or satiety feelings [27] [25]. Overweight patients lost weight during a short follow-up period of 4 months with an oral restriction device [28], however data from long-term follow-ups are not available. Two other approaches to prevent obese patients from overeating were wiring of jaws and intermaxillary fixation. [29] [30]. Both procedures achieved significant initial weight loss, but when the devices were removed weight was regained [31]. Another group studied a tongue patch for impeding swallowing combined with a low-calorie diet. Within the very small number of patients available for follow-up, 84% of patients maintained their initial weight loss during 3 months to 4 years [32] with high drop-out rates.

The change of eating behaviour translated into sustained weight loss in the consecutive months in the second phase during long-term follow-up, where the patients continuously lost weight without using the device. During long-term follow-up, all of the patients in our study significantly lost weight in a range defined as effective program [33].

Complex neurophysiological changes during cognitive control and unusual eating patterns of individuals have been identified as strong inducers of weight regain. Our approach to help overweight and obese patients to lose weight thus used this knowledge to change their behaviour and allow them to eat what they pleased. Our patients were not simply forced to eat slowly, but given the opportunity to savour [33] and indulge each and every bite. Thus, the two competing goals—namely, the aspiration to diet induced by cognitively inhibiting chronic self-control and the desire to eat with pleasure seem reconcilable by the use of this simple medical device.

This study has several limitations, such as the small number of 6 patients, the retrospective design of long-term follow-up with limited phenotypic data at inclusion and the long recruitment period as well as the lack of a control arm. We cannot exclude the possibility that patients have been included in this study who previously failed to reduce weight because of cravings caused by the sensory deprivation of monotonous diets.

Thus, a more powerful study is now conducted with a large number of patients in a prospective, randomised, controlled multicentre trial. This is to prove the concept and the underlying mechanism. Since the majority of patients will regain weight after conventional dieting, our approach may prove a serious alternative treatment for obesity in obese and overweight patients for whom evidence-based options have failed.

## Supporting information

S1 File TREND statement. (TIF)

S2 File English translation of the German Study protocol. (PDF)

S3 File Approval of the local ethics committee. (TIF)

S1 Protocol German Study protocol. (DOCX)
