# Supplementary material for: Persistent weight loss with a non-invasive novel medical device to change eating behaviour in obese individuals with high-risk cardiovascular risk profile
Source: PLoS One. 2017 Apr 12;12(4):e0174528. doi: 10.1371/journal.pone.0174528 (PMC5389612; doi:10.1371/journal.pone.0174528)
Supplement: S2 File — (PDF) [file pone.0174528.s002.pdf]

Study protocoll for a retrospective analysis of a prospective trial to plan a second prospective study

Complete title of the study

Development of a „Genusstrainers“ (engl. trainer for pleasure) for permanent weight reduction in obesity and overweight

1. Investigator

Principal investigator:
Dr. med. Peter von Seck
Rheinstraße 31,
65185 Wiesbaden
0611 /300755

Responsible for analysis:
PD Dr. med. Caroline Schmidt-Lucke
MEDIACC GmbH
Medizinisch-academische Forschungsberatungsgesellschaft mbH
Gneiststr. 4, 14193 Berlin
49 30 20847349
caroline.schmidt-lucke@mediacc.de

Other investigators:

PD Dr. med. dent. F. M. Sander
Sander & Klee Zahnärzte für Kieferorthopädie
Vilbeler Landstr. 3-5
60386 Frankfurt am Main
069 – 94 221 130
m.sander@kfo-klee.de

Frau Kerstin Amelung-Knur
Arzthelferin, Praxis Dr. med. Peter von Seck
Rheinstraße 31,
65185 Wiesbaden
0611 /300755

Frau Litsch
Arzthelferin, Praxis Dr. med. Peter von Seck
Rheinstraße 31,
65185 Wiesbaden
0611 /300755

Raimund H. Maurus
Medizintechniker und Informatiker in der Praxis Dr. med. Peter von Seck
Im Rheinblick 38,
D 55411 Bingen

Doctoral candidates (n. n., will be chose after approval of the ethic committee)

2. Background

Problem

The prevalence of the chronic disease overweight is around 35% and of obesity around 11% with similar numbers for Germany with 37 and 16%. The prevalence of this epidemic disease is increasing worldwide with the consequent quantities like impairment of well-being, quality of life, severe concomitant diseases, early retirement and increased mortality. This contrasts to the decreasing prevalence of other diseases. Current evidence-based interventional studies do not lead to the desired persistent weight loss.

Epidemiology and definition Epidemiologie und Definition

In 2015, 1.5 billion people will be overweight or obese. Overweight and obesity are associated with increased mortality. This disease is one of the five risk factors that most often account for worldwide mortality with estimated 3.4 billion death per year (August 2014).

Overweight and obesity most often are defined over the so-called body-mass index (BMI). Other measures are waste circumference or the relation of waist / hip ratio to identify abdominal fat. Abdominal fat in man and serves to identify increased health risks. Prevalence of overweight (BMI 25 to 30) in the German population lives around 37% (worldwide 35%) and of obesity (BMI >30) are around 16%, (worldwide 11%). Increasing over the last years. With increasing age, prevalence increases in both genders, with around 50% of men and 33% of women aged of 55 to 60 overweight or obese. In Germany, 40 million people are overweight. 35% (32 million) and 13% (12 million) of the working population are overweight or obese. In the working population (aged 18 to 40 and 65 years), 11% (39 more than non-obese) or 22% (18% more than non-obese) will call sick. As the WHO noted, these data warrant the considerable need for action and therapy to stop the increasing prevalence.

Direct and indirect costs

To be able to calculate the costs and to compare diseases, two key figure are calculated, derived from age of the patient at onset of disease, life expectancy of the person and extend of disability. Following the Global Burden of Disease (GBD), quality of life is defined as negative index of disability defining “Disability-Adjusted Life Year” (DALY). In contrast to other diseases (cardiovascular new plater) costs have been increasing for overweight and obesity worldwide by more than 30%. High BMI is a leading risk factor (6/25) for DALYs worldwide. All components of diet and physical inactivity that lead to overweight and obesity, make 10.2% of DALYs.

In addition to the direct costs that result from a disease (medical treatment, prevention, rehabilitation or nursing activities), considerable indirect costs result for the community. These arise from reduced working ability and early retirement. Patients with obesity have additional costs, that result from stigmatisation with consequent academic or professional achievements, including exclusion from better paying jobs.

Evidence-based intervention programs

Overweight and obesity can be prevented through environmental and community measurements. For weight reduction and to stabilise reduced weight, basic measures like teachings, behavioural changes, physical activity and healthy eating with a deficit in energy form the basis in evidence-based weight reduction programs. The advantages of weight reduction, even by 10%, regarding mortality, general health and obesity-associated diseases are well documented. Weight reductions result in improvements of diabetes mellitus 2, hyperlipidaemia, arterial hypertension, cardiovascular risk profile and ovary function, furthermore of reduced breathlessness, improvement of quality of sleep, sleep apnoea, back and joined pain, as well as osteoarthritis and psychosocial components.

Regardless of the composition of the diet a daily deficit of energy of around 500 cal per day results in a weight reduction of around 1.5 to 4.5 kg over a longer period. Cost-effective is defined as measures is for weight reduction that costs less than US$50,000 / DALY. This is the case for all state and societal programs as well as for most diets and behavioural changes.
The high percentage of quitters (45%) of programs and the so-called yo-yo effect with weight regain after nearly all programs are highly problematic. Current prevention therapies are characterised by a disproportional weight regain after interventions. This is especially the case for elderly patients that are not used to weight reduction programs including sufficient physical activity. This leads to constant weight gain with increasing age of the community and the individual.

Factors that influence the quantity of food include the quantity and variety of the food offered, visual and anticipatory stimuli and other behavioural patterns as well as satiety. Satiety is a complex process that is mediated by diverse short, middle and long acting mediators in the hypothalamus. Different brain areas integrate these complex neuronal and hormonal signals and thus influence food intake and other behavioural patterns or the emotional state.

The generator of the idea is a position that practices for around 50 years trying to make his patients lose weight with evidence-based programs. He noticed that the majority of patients, despite strong desire and recurrent interventions, continually gained weight over decades. From his passion as nature enthusiasts and wild farmer who tried to find a natural solution. From his practical experience over many years that diets, changing composition of food counting calories would lead to weight regain, he tried to find a new interventional approach. Despite knowing that chewing food longer to evoke the feeling of satiety the majority will not change their habit in real life.

Innovative approach

From the observation of many years, that patients will lose weight after tooth extractions and after an involuntary self-experiment the deal was born to include spaces in between teeth. The generator of the idea developed splints to reduce that chewing surface. This concept has been registered as patents in 2012.

He noticed after initially using these splints, that there was no weight regain and a preference towards food with less fat and higher quality resulting in the feeling of enjoyment whilst eating. Thus named splints Genusstrainer (enjoyment trainer).

The striking weight reduction of the innovator and his befriended patients led to high demand. This is the reason why a prospective clinical randomised trial is to be started. For this conception the data of the volunteers around the innovator shall be evaluated by a formal scientific analysis.

This first observational study should form the basis to define and standardise as well as concept the first clinical study to prove the concept of the Genusstrainers as medical device risk class I proving efficacy, safety and benefit.

Surplus studies being carried out and if yes, what was the result?
No/not applicable

References

1. WHO. Http://www.Who.Int/mediacentre/factsheets/fs311/en/. 2014
2. Dormenval V, Mojon P, Budtz-Jorgensen E. Associations between self-assessed masticatory ability, nutritional status, prosthetic status and salivary flow rate in hospitalized elders. *Oral diseases*. 1999;5:32-38
3. Murray CJ, Lopez AD. Measuring the global burden of disease. *N Engl J Med*. 2013;369:448-457
4. Mensink GB. Übergewicht und adiposits in deutschland. *Bundesgesundheitsblatt, Gesundheitsforschung, Gesundheitsschutz*. 2013;56:786-794
5. Bundesamt S. Statistisches jahrbuch *https://www.destatis.de/DE/Publikationen/StatistischesJahrbuch/StatistischesJahrbuch2014.html;jsessionid=FE08C762C70F61A06D2EB29E732AE56E.cae4*. 2014
6. Lehnert T, Sonntag D, Konnopka A, Riedel-Heller S, Konig HH. The long-term cost-effectiveness of obesity prevention interventions: Systematic literature review. *Obesity reviews : an official journal of the International Association for the Study of Obesity*. 2012;13:537-553
7. Wirth A, Wabitsch M, Hauner H. The prevention and treatment of obesity. *Deutsches Arzteblatt international*. 2014;111:705-713
8. Cecchini M, Sassi F, Lauer JA, Lee YY, Guajardo-Barron V, Chisholm D. Tackling of unhealthy diets, physical inactivity, and obesity: Health effects and cost-effectiveness. *Lancet*. 2010;376:1775-1784
9. Tobias D, Pan A, Hu FB. Bmi and mortality among adults with incident type 2 diabetes. *N Engl J Med*. 2014;370:1363-1364
10. Mozaffarian D, Hao T, Rimm EB, Willett WC, Hu FB. Changes in diet and lifestyle and long-term weight gain in women and men. *N Engl J Med*. 2011;364:2392-2404

3. Hypothesis

The aim is to test the main hypothesis that using the Genusstrainers will lead to a persistent weight reduction.

3.1. What is expected?

From this analysis of the first volunteers the hypothesis shall be tested that
- there was a continuous increase of weight and of the visceral fat over the last 10 years related to the baseline weight when they splints were first used

- there will be a weight reduction and reduction of the visceral fat and now weight regain, even after stopping using the splints.

3.2. Aim of the study

Primarily the efficacy of the splints shall be proven. The primary endpoint is weight reduction after using the splints. Secondary endpoints regarding mode of action, safety and benefit shall be analysed, if the existing documentation and is sufficient.
Hereafter, the splints shall be standardised regarding the construction and further specified. This will form the basis for the development and certification of the medical device (MD) risk class I and consequent testing in a clinical trial.

It will be the aim of this retrospective analysis of the current observational study to obtain data so that a study protocol can be derived from this including a proof of mechanism for a then certified MD.
I large-scale prospective randomised controlled trial shall be planned to prove the main hypothesis and further to be identified secondary hypotheses to test the splints as medical device.

4. Targets

Following the main hypothesis data shall be analysed regarding the following influencing and target variables:

<u>Baseline values:</u>

– age (in years) and time point 0
– gender
– date of time point 0 and of the earliest documented weight
– weight and waist circumference at time point 0
– height at time point 0
– BMI at time point 0
– categories: BMI > 25, BMI > 30, BMI > 35 .0
– duration of previous attempts to reduce weight
– development of weight and waist circumference over the last years
– number of previous attempts to lose weight
– type of weight reduction programs
– change of medication and inclusion (one month follow-up periods)

– Concomitant diseases
– metabolic syndromes (yes / no)
– Diabetes mellitus (y/n)
– Duration Diabetes mellitus (only in DM patients)
– family history for premature cardiovascular events; m < 55 years , w < 65 years, (y/n)
– current smokers , (y/n)
– hyperlipoproteinaemia (diabetes LDL > 70 mg / dl, non-diabetes> 100 mg /dl OR statin therapy), (y/n)
– art. Hypertension, (y/n)
– number of cardiovascular risk factors (including: family history; m < 55 years , w < 65 years , smoking , hyperlipoproteinaemia (diabetes LDL > 70 mg / dl, non-diabetes> 100 mg /dl OR statin therapy), art. hypertension , Diabetes mellitus)

- orthopaedic problems (y/n)
  type of orthopaedic problem
- social economic status, training, income (rough estimation), status employee, retired

Target variables

- **weight, BMI and waist circumference after using the MD**
- duration of follow-up (in month all years)
- number of follow-up visits
- date of last follow-up visit

- Concurrent medication
- change of medication during follow-up (y/n), which?

- Laboratory findings:
- HLP, HBA1C, hs-CRP, BB,

- Sports or physical activity
- adherence of using the MD
- subjective details regarding the MD
- change of eating behaviour
- psychologic statement of the personnel of the medical practice of Dr. von Seck
- change of quality of life and physical performance

Time point 0 is the visit at which the patient decided to use the MD.

5. Study design

This is a retrospective analysis to be carried out after obtaining written informed consent of the volunteers that their data shall be used for scientific research (see appendix).

Choice of data
See Number 4.

Origin of data
All data shall be extracted from patients records by two independent nurses or the lead investigator. In doubt there will be a consultation and consensus building. In case that one of the volunteers is not a patient of the medical Praxis, data will be obtained via telephone and /other rackets provided by the patient will be reviewed.

Acquisition of data
Data will be acquired in electronic database and pseudomised. Thereafter, data will be analysed statistically with SPSS. Prior to analysis they will be examined for completeness, accuracy and credibility.

Data safety
All databanks will be secured with passwords against unauthorised access. Electronic transmission of any study contents or data will be done with password encryption. The password key, at least eight characters and/or digits through another medium (SMS telephone or post). It shall be changed every other four weeks. Only persons subsidised under number one shall have access to personal data. Uncoded data shall only be seen via Praxis personnel of the medical Praxis of Dr von Seck and Sander.

Data analysis

All data that will be quiet for this analysis, shall only serve to answer the main question. No other data shall be acquired analysed for any other purpose. All data shall be acquired and analysed according to GCP, following the declaration of Helsinki and by complying to all EU regulations and the German legislation.

6. Number of patients / volunteers

Five volunteers (+ the inventor) have used the MD. Data from these volunteers shall be analysed.

7. Statistics

This is a retrospective analysis. Most likely, data of some variables will be missing. Data from weight courses/visceral fat from the last 10 years shall be analysed. Most likely only weight courses might be documented. This will only have been done according to clinical necessity. There will be no standardisation regarding time points.
All continuous variables shall be checked for normal distribution with the Kolmogorov-Smirnov test. Data shall be expressed as mean expressed as mean ± standard deviation. Normally distributed variables shall be analysed with the two-sided t-test. Not normally distributed variables shall be analysed with the Mann-Whitney-U Test. Comparison of categorical variables shall be generated with the Pearson χ^2-test and the Fishers exact test. The linear regression analysis shall be used to identify independent predictors for weight reduction in the follow-up period. Is not expected that these tests shall be used regarding the small number of volunteers. Statistical significance shall be assumed if a null hypothesis can be rejected at $p \leq 0.05$. All statistical analysis shall be performed with SPSS 20 ®.

8. Inclusion and exclusion criteria

Inclusion criteria

1. age: 18 to 95 years
2. gender male and female
3. ability to consent
4. written informed consent
5. exploit of the established evidenced-based options for weight reduction
6. receiving an MD
7. at least two documented visits after receiving the MD

Exclusion criteria for the analysis

1. weight reduction of at least 5% during the last six weeks before receiving and using the MD
2. wasting disease
3. taking medication that affects weight (steroids, psychoactive medication, diuretics , etc.)
4. pregnancy or breastfeeding mothers
5. reduce compliance or psychological disorders
6. inability to consent
7. participating in another clinical study

9. Data privacy protection

All analysis that goes beyond clinically indicated and Sämtliche Auswertungen über die klinische indizierten und all investigations in humans are subject to the current legislation, rules and directives, the ethical guidelines of the World Medical Association from 1996, the Germans medical associations professional code of conduct as well as the Data Protection Act.

Patients shall be asked prior to the analysis via telephone about their consent to participate. In case of agreement they shall be provided with a consent form. Only after receiving the informed consent shall the data be analysed.

All analysis shall be performed pseudomised with the code that consists of characters and digits. Only the practitioner and the assisting personnel shall have access to the original data from where the patients may be re-identified.

Electronic databases shall only be transmitted encrypted. The code, consisting of a combination of at least eight characters and digits shall always be communicated through another medium.

10. Date and signature of the lead investigator
