# Supplementary material for: Persistent weight loss with a non-invasive novel medical device to change eating behaviour in obese individuals with high-risk cardiovascular risk profile
Source: PLoS One. 2017 Apr 12;12(4):e0174528. doi: 10.1371/journal.pone.0174528 (PMC5389612; doi:10.1371/journal.pone.0174528)
Supplement: S1 Protocol — (DOCX) [file pone.0174528.s004.docx]

**Prüfplan für eine retrospektive Auswertung zur Planung einer prospektiven Studie**

**Vollständiger Titel der Studie**

**Entwicklung eines Genusstrainers zur dauerhaften Gewichtsreduktion bei Übergewicht und Adipositas**

**1. Prüfer**

Studienleiter:

Dr. med. Peter von Seck

Rheinstraße 31,

65185 Wiesbaden

0611 /300755

Verantwortlich für die Auswertung:

PD Dr. med. Caroline Schmidt-Lucke

MEDIACC GmbH

Medizinisch-academische Forschungsberatungsgesellschaft mbH

Gneiststr. 4, 14193 Berlin

49 30 20847349

caroline.schmidt-lucke@mediacc.de

Sonstige Untersuchende:

PD Dr. med. dent. F. M. Sander

Sander & Klee Zahnärzte für Kieferorthopädie

Vilbeler Landstr. 3-5

60386 Frankfurt am Main

069 – 94 221 130

m.sander@kfo-klee.de

Frau Kerstin Amelung-Knur

Arzthelferin, Praxis Dr. med. Peter von Seck

Rheinstraße 31,

65185 Wiesbaden

0611 /300755

Frau Litsch

Arzthelferin, Praxis Dr. med. Peter von Seck

Rheinstraße 31,

65185 Wiesbaden

0611 /300755

Raimund H. Maurus

Medizintechniker und Informatiker in der Praxis Dr. med. Peter von Seck

Im Rheinblick 38,

D 55411 Bingen

Doktorandin (noch nicht bekannt, Option wird nach positivem Votum durch die EK ausgeschrieben)

**2. Hintergrund**

**Problemstellung**

Die Prävalenz der chronischen Erkrankung Übergewicht liegt weltweit bei 35% und die der Adipositas bei 11%, entsprechend für Deutschland bei 37% und 16%. Weltweit nimmt die Prävalenz dieser inzwischen epidemischen Erkrankung mit ihren vielfältigen gesundheitlichen Problemen wie Störungen des Wohlbefindens, der Lebensqualität, gravierenden Folgeerkrankungen, vorzeitiger Berentung und erhöhte Mortalität gegen den Trend der Prävalenz der übrigen Erkrankungen stetig zu. Die derzeitigen evidenzbasierten Interventionsstudien führen bei den meisten Individuen nicht zu dem gewünschten kontinuierlichen Gewichtserhalt.

**Epidemiologie und Definition**

Es werden in 2015 1,5 Milliarden Menschen übergewichtig oder adipös sein. Übergewicht und Adipositas sind mit einer erhöhten Sterblichkeit assoziiert, wobei diese Erkrankung inzwischen der fünfthäufigste Risikofaktor der weltweiten Mortalität mit geschätzten 3,4 Millionen Todesfällen jährlich ist (Stand August 2014).

Übergewicht und Adipositas werden zumeist über den body mass index (BMI) definiert. Außerdem können Bauchumfang und das Verhältnis Bauch / Hüfte als Maß für abdominelles Fett herangezogen werden. Abdominelles Fett ist häufiger bei Männern und dient zur Identifizierung eines erhöhten gesundheitlichen Risikos.

Die Prävalenz von Übergewicht (BMI 25 bis 30) in der deutschen Bevölkerung liegt bei 37% (weltweit 35%) und die der Adipositas (BMI <30) bei 16% (weltweit 11%), wobei die Tendenz in den letzten Jahren steigend ist. Mit zunehmendem Alter nimmt die Prävalenz in beiden Geschlechtern zu, so dass ab einem Alter von 55 bis 60 50% der Männer und bei Frauen 33% übergewichtig und adipös sind. In Deutschland sind also 40 Mio übergewichtig und 14,4 Mio Einwohner adipös. 35% (32 Mio) und 13% (12 Mio) der Erwerbstätigen sind übergewichtig bzw. adipös. Bei der als erwerbstätig eingestuften adipösen Bevölkerung (Altersgruppen 18 bis 40 und 40 bis 65 Jahre) sind 11% (39% mehr als nicht-adipös) bzw. 22% (18% mehr als nicht-adipös) als krank gemeldet. Hieraus ergibt sich der erhebliche Handlungs- und Therapiebedarf, wobei die WHO fordert der zunehmenden Prävalenz zu stoppen.

**Direkte und indirekte Kosten**

Um die Belastungen durch eine Krankheit zu berechnen und vergleich zu können, werden das Alter bei Einsetzen der Erkrankung, die Lebenserwartung der betroffenen Person und das Ausmaß der Behinderung zu Grunde gelegt. Hiernach gibt es 2 Kennzahlen. Nach der Globalen Krankheitslast-Studie (Global Burden of Disease – GBD) wird  für das Maß „Lebensqualität“ ein negativer Behinderungsindex, der bei hohen Werten eine niedrige Lebensqualität beschreibt: das behinderungsbereinigte Lebensjahr (Disability-Adjusted Life Year, DALY) definiert.

Die Kosten, die durch Übergewicht und Adipositas verursacht werden sind nach den aktuellen Berichten der WHO, entgegen dem Trend der übrigen führenden Erkrankungen (kardiovaskulär, Neoplasien) um mehr als 30% gestiegen. Ein hoher BMI ist ein führender Risikofaktor (6 von 25) für DALYs weltweit. Weltweit machen alle Komponenten der Diät und körperlichen Inaktivität, die zu Übergewicht und Adipositas führen 10,2% der DALYs aus. Neben den direkten Kosten, die sich aus einer Erkrankung ergeben (medizinische Heilbehandlung, Präventions-, Rehabilitations- oder Pflegemaßnahme), entstehen erhebliche indirekte Kosten für die Gesamtbevölkerung. Diese entstehen durch reduzierte Arbeitsfähigkeit, vorzeitige Berentung. Für die betroffenen kommen noch weitere Kosten hinzu, das die Adipositas zu Stigmatisierung führt mit konsequenten schlechten akademischen oder beruflichen Leistungen, inklusive dem häufigen Ausschluss von besser bezahlten Arbeitsstellen.

**Evidenzbasierte Interventionsprogramme**

Übergewicht und Adipositas können vermieden werden durch Umwelt- und gesellschaftliche Maßnahmen. Zur Gewichtsreduktion und zur Stabilisierung eines reduzierten Gewichts werden individualisiert die Basismaßnahmen Schulung, Verhaltens­änderungen, körperliche Aktivität und Ernährung mit einem Energiedefizit in wissenschaftlich evaluierten Gewichtsreduktionsprogrammen empfohlen. Die Vorteile einer Gewichtsreduktion, selbst einer moderaten um 10%, auf Mortalität, allgemeine Gesundheit und Adipositas-assoziierte Erkrankungen sind gut belegt. Beabsichtigte Gewichtsreduktion resultiert in Verbesserungen von DM 2, HLP, Hypertension, kardiovaskulärem Risiko und Ovarialfunktion. Außerdem verbessern sich Luftnot, Schlafqualität, Schlafapnoe Rücken- und Gelenkschmerzen sowie Osteoarthritis und die psychosozialen Komponenten.

Unabhängig von der Zusammensetzung bewirkt eine Reduktionskost mit einem täglichen Energiedefizit von ca. 500 kcal / Tag evidenzbasiert eine Gewichtsreduktion von etwa 1,5 bis 4,5 kg über einen längeren Zeitraum.

Als kosteneffektiv werden Maßnahmen zur Gewichtsreduktion angesehen, bei denen weniger als 50 000 US Dollar / DALY anfallen. Dies gilt für sämtliche staatliche und gesellschaftliche Programme, sowie für die meisten Diäten und Verhaltensänderungen.

Problematisch sind jedoch zum einen der hohe Anteil der Ausscheider aus den Programmen (bis um die 45%) und zum anderen die im Volksmund als Jo-Jo-Effekt bezeichnete deutliche Gewichtszunahmen nach fast allen Programmen. Gängige Präventionen und Therapie sind vor Allem durch eine unverhältnismäßige Gewichtszunahme nach der Intervention gekennzeichnet. Dies gilt insbesondere für ältere Patienten, die gewohnheitsbedingt nicht an Programmen mit ausreichender körperlicher Betätigung teilnehmen. Dies führt bei vielen Menschen und mit zunehmendem Alter zu eine kontinuierlichen Gewichtszunahme der Bevölkerung und des Individuums.

Zu den Faktoren, die dazu beitragen, wie viel Nahrung aufgenommen wird, gehören Menge und die Abwechslung der angebotenen Nahrung, visuelle, optische, antizipatorische Stimuli und andere Verhaltensmuster sowie das Sättigungsgefühl. Das Sättigungsgefühl ist ein komplexer Vorgang, das über verschiedene kurz-, mittel- und langwirksame Mediatoren im Hypothalamus vermittelt wird. Unterschiedliche Hirnarealen integrieren die komplexen neuronalen und hormonellen Signale und beeinflussen damit die Nahrungsaufnahme und andere Verhaltensmuster oder den Gemütszustand.

Der Ideengeber ist seit 50 Jahren praktizierender Arzt und Internist und hat vielen seiner Patienten evidenzbasiert bereits mehrere Gewichtsreduktionsprogramme verordnet. Auffällig war ihm, dass ein Großteil der Patienten, trotz starken Willens und rezidivierender Interventionen, über Dekaden langfristig kontinuierlich zunahm. Aus seiner Passion als Naturliebhaber und Wildfarmer hat er durch genaue Beobachtung den Menschen als „Seelenesser“ im Gegensatz zum Tier als „Instinktesser“ unterschieden, und versucht hierfür eine Lösung zu finden. Er versuchte aus der Beobachtung, dass wildlebende Säugetiere nie fetter sind, als für sie nützlich ist, eine Lösung für seine Patienten zu finden. In dem praxiserprobten langjährigen Wissen, dass Diäten mit Ernährungsergänzungen, Umstellung der Zusammensetzungen oder Kalorienzählen zu einer erneuten Gewichts­zunahme führen, hat er sich um einen neuen Interventionsansatz bemüht.

Der Ansatz, das Essen länger zu kauen, damit sich früher ein Sättigungsgefühl einstellt, scheitert in praxi an den Mahlzeiten, die dann doch nicht lange genug gekaut wurden. Dies verhindert dann die Interaktion zwischen Verdauungstrakt und den für das Sättigungsgefühl verantwortlichen Hirnareale.

**Lösungsansatz**

Aus der ebenfalls über Jahre gewonnen Beobachtung, dass Patienten nach Gebisssanierungen langanhaltend Gewicht verloren und nach einem unfreiwilligen Selbstversuch mit deutlichem Gewichtsverlust entstand die Idee einen Abstandhalter zwischen die Zähne beim Essen einzubauen. Mit zahnärztlichen Freunden hat der Ideengeber eine Schiene entwickelt, die die Kaufläche reduziert.

Dieses Konzept ist 2012 zum Patent angemeldet worden.

Auffällig waren wohl insbesondere das Fehlen der erneuten Gewichtszunahme, die Änderung der Präferenz gegenüber fettarmen und höherwertigen Speisen und einem Wiedereinstellen eines neuen „Genussgefühls“. Daher wird diese Schiene auch Genusstrainer genannt.

Die auffällige Gewichtsreduktion des Ideengebers und vier seiner Freunde führt zu einer hohen Nachfrage, die nun im Rahmen einer klinischen Studie mit einem zu konzipierendem Studiendesign bedient werden soll. Für die Konzeption einer prospektiven Studie sollen zunächst die Daten der Freiwilligen aus dem Bekanntenkreis des Ideengebers wissenschaftlich ausgewertet werden.

Diese erste Anwendungsbeobachtung soll Grundlage für das Definieren und Standardisieren sowie der Konzeption einer ersten klinischen Studie zur Prüfung des Genusstrainers als Medizinprodukt der Risikoklasse I auf Wirksamkeit, Sicherheit und Nutzen sein.

**Wurden schon ähnliche Studien durchgeführt und wenn ja mit welchem Ergebnis?**

Nein / entfällt

**Literaturliste**

1. WHO. <Http://www.Who.Int/mediacentre/factsheets/fs311/en/>. 2014

2. Dormenval V, Mojon P, Budtz-Jorgensen E. Associations between self-assessed masticatory ability, nutritional status, prosthetic status and salivary flow rate in hospitalized elders. *Oral diseases*. 1999;5:32-38

3. Murray CJ, Lopez AD. Measuring the global burden of disease. *N Engl J Med*. 2013;369:448-457

4. Mensink GB. Übergewicht und adiposits in deutschland. *Bundesgesundheitsblatt, Gesundheitsforschung, Gesundheitsschutz*. 2013;56:786-794

5. Bundesamt S. Statistisches jahrbuch *https://*[*www.destatis.de/DE/Publikationen/StatistischesJahrbuch/StatistischesJahrbuch2014.html;jsessionid=FE08C762C70F61A06D2EB29E732AE56E.cae4*](http://www.destatis.de/DE/Publikationen/StatistischesJahrbuch/StatistischesJahrbuch2014.html;jsessionid=FE08C762C70F61A06D2EB29E732AE56E.cae4). 2014

6. Lehnert T, Sonntag D, Konnopka A, Riedel-Heller S, Konig HH. The long-term cost-effectiveness of obesity prevention interventions: Systematic literature review. *Obesity reviews : an official journal of the International Association for the Study of Obesity*. 2012;13:537-553

7. Wirth A, Wabitsch M, Hauner H. The prevention and treatment of obesity. *Deutsches Arzteblatt international*. 2014;111:705-713

8. Cecchini M, Sassi F, Lauer JA, Lee YY, Guajardo-Barron V, Chisholm D. Tackling of unhealthy diets, physical inactivity, and obesity: Health effects and cost-effectiveness. *Lancet*. 2010;376:1775-1784

9. Tobias D, Pan A, Hu FB. Bmi and mortality among adults with incident type 2 diabetes. *N Engl J Med*. 2014;370:1363-1364

10. Mozaffarian D, Hao T, Rimm EB, Willett WC, Hu FB. Changes in diet and lifestyle and long-term weight gain in women and men. *N Engl J Med*. 2011;364:2392-2404

**3. Hypothese**

Es soll die Haupthypothese geprüft werden, dass der Einsatz des Genusstrainers zu einer dauerhaften Gewichtsreduktion führt.

**3.1. Was wird erwartet?**

Aus der retrospektiven Auswertung der Daten der bisherigen Freiwilligen soll die Hypothese geprüft werden, dass

- es zu einer kontinuierlichen Zunahme des Gewichts und des viszeralen Fetts über etwa 10 Jahren im Vergleich zum Eingangsgewicht zum Zeitpunkt des Anpassens der Genussschiene,
- einer folgenden Gewichtsreduktion und des viszeralen Fetts mit Erhalt des Gewichts auch nach Beenden der Nutzungsphase des Genusstrainers

gekommen ist.

**3.2. Ziel der Studie**

Es soll primär die Wirksamkeit des Genusstrainers anhand des primären Endpunkts belegt werden. Der primäre Endpunkt ist die Gewichtsreduktion nach Benutzen des Genusstrainers. Begleitend sollen die sekundären Endpunkte zu Wirksamkeit, Sicherheit und Nutzen untersucht werden, sofern die vorliegenden Aufzeichnungen hierfür überhaupt geeignet sind.

Hiernach wird der Genusstrainer bezüglich seiner Konstruktion standardisiert und definiert. Dies wird die Grundlage sein für die Entwicklung und Zertifizierung eines Medizinprodukts (MP) für die Testung in einer klinischen Studie der Risikoklasse I durch den Hersteller.

Das Ziel der jetzt beantragten retrospektiven Studie ist es, Daten zu erhalten, auf Grund deren sich kurzfristig ein Prüfplan ableiten lässt für eine kleinere prospektive klinische Studie, mit der mögliche Wirkmechanismen eines dann für die Studie zertifizierten MP belegt werden können.

Mit den dort gewonnenen Daten soll dann mittelfristig eine größere prospektive randomisierte, kontrollierte Studie oder Beobachtungsstudien zur Prüfung der Haupthypothese und noch zu identifizierenden Nebenhypothesen erarbeitet werden, um den Genusstrainer als MP zu testen.

**4. Zielgrößen**

Entsprechend der Haupthypothese sollen Daten zu den folgenden Einfluss- und Zielvariablen erfasst werden:

Eingangsgrößen:

- Alter (in Jahren) zum Zeitpunkt 0
- Geschlecht
- Datum von Zeitpunkt 0 und von einem frühestmöglichen dokumentiertem Gewicht
- Ausgangsgewicht und Bauchumfang zum Zeitpunkt 0
- Körpergröße zum Zeitpunkt 0
- BMI zum Zeitpunkt 0 (kann ich auch mit einem Mausklick berechnen)
- Kategorie: BMI > 25, BMI > 30, BMI > 35 zum Zeitpunkt 0
- Zeitdauer von Abnahmeversuchen (ab wann begannen erste Gespräche über Notwendigkeit des Abnehmens
- Gewichtsverläufe und Bauchumfang der letzten Jahre
- Anzahl der vorangegangenen Abnahmeversuche
- Art der Abnahmeprogramme
- Wechsel der Medikation vor Einschluss (1 Monat Beobachtungszeitraum)
- Begleiterkrankungen
- Metabolisches Syndrom (ja / nein)
- Diabetes mellitus (j/n)
- Dauer Diabetes mellitus (nur wenn DM vorhanden)
- Familienanamnese für vorzeitige kardiovaskuläre Ereignisse; m < 55 Jahre, w < 65 Jahre, (j/n)
- aktueller Nikotinabusus, (j/n)
- Hyperlipoproteinämie (Diabetiker LDL > 70 mg / dl, Nicht-DM > 100 mg /dl ODER Statintherapie), (j/n)
- art. Hypertonus, (j/n)
- Anzahl kardiovaskulärer Risikofaktoren (hierzu zählen: Familienanamnese für vorzeitige kardiovaskuläre Ereignisse; m < 55 Jahre, w < 65 Jahre, aktueller Nikotinabusus, Hyperlipoproteinämie (Diabetiker LDL > 70 mg / dl, Nicht-DM > 100 mg /dl ODER Statintherapie), art. Hypertonus, Diabetes mellitus)
- Othopädische Probleme (j/n)
- Art orthopädisches Problem
- Soziökonomischer Status, Ausbildung, Einkommen (grobe 3er Einteilung), Angestelltenstatus, berentet

Zielvariablen

- **Gewichtsverläufe, BMI und Bauchumfang nach Einsatz des Genusstrainers**
- Länge der Nachbeobachtung (in Monaten oder Jahren)
- Anzahl der wahrgenommenen Nachbeobachtungsvisiten
- Datum der letzten Visite
- Begleitmedikation
- Wechsel der Medikation während der Nachbeobachtung (j/n), welche?
- Labor:
- HLP, HBA1C, hs-CRP, BB,
- Sportliche Aktivität
- Adhärenz zum Einsatz des Genusstrainers
- Subjektive Angaben zum Genusstrainer
- Änderung der Ernährungsgewohnheiten
- Psychologische Einschätzung durch Praxispersonal und Dr. von Seck
- Änderung der Leistungsfähigkeit und Lebensqualität

Zeitpunkt 0 ist die Visite, bei der der Patient zu einem Genusstrainer entschlossen hat.

**5. Untersuchungsdesign**

Es handelt sich um eine retrospektive Analyse der Daten nach erfolgtem schriftlichem Einverständnis durch die Freiwilligen. (Einverständnis zur Auswertung s. Anhang)

Auswahl der Daten

Siehe hierzu bitte Punkt 4.

Herkunft der Daten

Die Daten werden aus den Krankenunterlagen extrahiert durch zwei unabhängig voneinander arbeitende Praxisangestellte oder dem Studienleiter. Im Zweifelsfall erfolgt eine Rücksprache und Konsensbildung. Sofern ein Teilnehmer nicht Patient der Praxis ist, werden seine Daten telefonisch erfragt und / oder Unterlagen, die der Teilnehmer zur Verfügung stellt, eingesehen.

Erfassung der Daten

Die Daten werden in einer EXCEL-Datenbank erfasst und pseudonymisiert. Hiernach werden die Daten in SPSS zur statistischen Auswertung übertragen. Vor der Auswertung werden sie auf Vollständigkeit, Fehlerfreiheit, Glaubwürdigkeit überprüft.

Gewährleistung der Datensicherheit

Die Datenbanken werden durch Passwörter vor dem Einsehen durch Unbefugte geschützt. Bei elektronischer Übermittlung jeglicher Studieninhalte oder Daten wird eine passwortgeschützt Verschlüsselung eingehalten. Die Passworte Der Schlüssel, bestehend aus einer mindestens 8stelligen Buchstaben und Zahlenkombination, wird immer über ein anderes Medium (SMS, Telefon oder Briefumschlag) übermittelt und regelmäßig, mindestens alle 4 Wochen geändert. Nur die unter 1. aufgeführten Personen haben Zugang zu den unkodierten Daten. Die unkodierten Daten können nur von den Praxisangestellten der Praxen von Seck und Sander eingesehen werden.

Auswertung von Daten

Alle Daten, die im Rahmen dieser Auswertung erfasst werden, dienen lediglich der Beantwortung der Hauptfragestellung. Es werden keine darüberhinausgehenden Daten erfasst oder zu anderem Zwecken ausgewertet. Hierbei werden die Daten CGP-konform erfasst und ausgewertet, entsprechend den ethischen Grundsätzen der Deklaration von Helsinki und unter Einhaltung aller EU-Regularien und der deutscher Gesetzgebung.

**6. Anzahl der Patienten / Probanden**

Es haben 5 Teilnehmer freiwillig sich einen Genusstrainer benutzt. Die Daten diesen Freiwilligen sollen ausgewertet werden.

**7. Statistik**

Es handelt sich bei der Studie um eine retrospektive Auswertung. Hierbei ist zu erwarten, dass die zu evaluierenden Variablen unvollständig sind. Es werden vorhanden Gewichtsverläufe / viszerales Fett aus den letzten 10 Jahren untersucht. Sicher sind nur die Gewichtsverläufe dokumentiert. Diese auch nur entsprechend den klinischen Notwendigkeiten. Eine Standardisierung bezüglich der Zeitpunkte liegt nicht vor.

Sämtliche kontinuierlichen Variablen werden mittels Kolmogorov-Smirnov auf Normalverteilung untersucht. Die Daten werden als Mittelwert ± Standardabweichung dargestellt. Normalverteilte Variablen werden dann mit dem t-Test (zweiseitig) analysiert. Nicht-normalverteilten Variablen und werden mit dem Mann-Whitney-U Test untersucht. Vergleiche zwischen zwei kategorischen Variablen wurden mit dem Pearson χ^2^-Test untersucht. Der bivariate Spearman-Rho-Korrelationskoeffizient wird für ordinalskalierte Parameter berechnet. Mittels linearer Regressionsanalyse kann untersucht werde, welche Größen unabhängige Prädiktoren für die Gewichtsreduktion im Nachbeobachtungszeitraum sind. Der Einsatz dieses Tests wird bei der geringen Fallzahl nicht erwartet. Eine statistische Signifikanz wird angenommen, wenn die Null-Hypothese mit einem Signifikanzniveau von P ≤ 0.05 abgelehnt werden kann. Für die Auswertung wird SPSS 20 ® benutzt.

**8. Ein- und Ausschlusskriterien**

**Einschlusskriterien**

1. Alter: 18 bis 95 Jahren
2. Geschlecht: Männer und Frauen
3. Einwilligungsfähigkeit
4. Unterschriebene Einwilligungserklärung nach Patientenaufklärung
5. Ausschöpfen der etablierten, evidenzbasierten Optionen der Gewichtsreduktion
6. Erhalt eines Genusstrainers
7. Mindestens 2 dokumentierte Visiten nach Erhalt eines Genusstrainers

**Ausschlusskriterien für die Auswertung**

1. Abnahme von > 5% in den 6 Wochen vor Erhalt und Benutzung eines Genusstrainers
2. Konsumierende Erkrankung
3. Einnahme von gewichtsbeeinflussenden Medikamenten (Steroide, Psychopharmaka, Diuretika, etc.)
4. Schwangerschaft oder stillende Mütter
5. Mangelhafte Compliance oder psychische Störungen
6. Nichteinwilligungsfähigkeit
7. Teilnahme an einer anderen klinischen Untersuchung

**9. Datenschutz**

Sämtliche Auswertungen über die klinische indizierten und erfolgten Untersuchungen am Menschen erfolgen unter Beachtung der bestehenden Gesetze, Vorschriften und Richtlinien, der Deklaration des Weltärztebundes über biomedizinische Forschung am Menschen von 1996, die Berufsordnung der deutschen Ärzte sowie das Datenschutzgesetz.

Die Patienten werden vor der Auswertung ihrer Daten telefonisch um ihr Einverständnis gebeten. Im positiven Fall bekommen Sie eine Einverständniserklärung zugeschickt. Erst nach Erhalt des Einverständnisses werden die Daten ausgewertet.

Die Auswertung erfolgt pseudonymisiert mit einem Code, der aus einer Buchstaben- und Zahlenkombination besteht. Lediglich der behandelnde Arzt und die Praxishilfen haben Zugang zu den Originaldaten, aus denen sich die Identität der Patienten herleiten lässt.

Eine elektronische Datenbank wird nur verschlüsselt verschickt. Der Schlüssel, bestehend aus einer mindestens 8stelligen Buchstaben und Zahlenkombination, wird immer über ein anderes Medium kommuniziert.

**10. Datum und Unterschrift des Studienleiters**

**Anhang**

**Inhalt**

Ablauf des bisherigen Vorgehens

Herstellung und Einsatz des Genusstrainers

Mögliche Erklärung des Wirkmechanismus

Einschätzung potenzieller Risiken aus kiefernorthopädischer Sicht und individuelle Aufklärung

Vordruck Patienteninformation

Vordruck Einverständniserklärung

**Ablauf des bisherigen Vorgehens**

Die Freiwilligen haben sich um diesen Genusstrainer bemüht und sind über das experimentelle Vorgehen von ärztlicher Seite im persönlichen Gespräch aufgeklärt worden. Sie haben sich aus dem Bekanntenkreis des Innovators rekrutiert.

Das Aufklärungsgespräch umfasste:

# Informationen zum natürlichen Verlauf der Erkrankung mit kontinuierlicher Gewichtszunahme trotz mehrfacher evidenzbasierter Interventionen

# vollständige Nennung aller Optionen, gegebenenfalls einschließlich der Möglichkeit, diese Intervention nicht wahrzunehmen,

# Wahrscheinlichkeiten für Erfolg, Nichterfolg und Schaden zu den anstehenden medizinischen

# Interventionen anhand eines Einzelbeispiels,

# patientenrelevanter Zielparameter: Gewichtsverlust

- sich beim Essen Zeit zu lassen
- Trinken vor und nach dem Essen,
- Training des verstärkten Genuss,
- vorgewärmte Keramikteller um die Mahlzeit warm zu halten
- Bestecke werden beim Kauen abgelegt
- 2 kleinere Portionen nehmen
- intensiv darauf hingewiesen: keine Diät einhalten.

# das Fehlen der Evidenz für den Genusstrainer

# für diagnostische Maßnahmen: gelegentliche Anrufe und Mitteilen des Gewichts durch den Freiwilligen, bei klinisch indizierter Visite in der Praxis wiegen.

# Sofortige Mitteilung an den Kieferorthopäden, bei Problemen, die sich durch die Schiene ergeben

# Engmaschige Kontrolle von Blutzucker bei einem Diabetiker und ggf. konsequente Reduktion der Insulindosis, Aufsuchen des behandelnden Hausarztes

Eine schriftliche Begleitinformation wurde den Freiwilligen mitgegeben.

**Herstellung und Einsatz des Genusstrainers**

Zweiteilige Zahnschienen, wie sie als sogenannte „Knirschschienen“ von Kieferorthopäden angefertigt werden, wurden zu Genusstrainern modifiziert. Hierbei handelt es sich um eine „Sonderanfertigungen“ im Sinne des § 3 Nr. 8 MPG. Es wurde nach standardisiertem Protokollen und bestem zahnärztlichen Wissen und höchster Sorgfalt vorgegangen. Das Vorgehen wird im Folgenden beschrieben.

Vor Anpassen einer Schiene wurden die Freiwilligen ausführlich von dem Kiefernorthopäden aufgeklärt. Sämtliche Kosten wurden von den Freiwilligen selbstständig getragen. Weder ärztliche noch zahnärztliche Leistungen sind gegenüber den jeweiligen Krankenkassen abgerechnet worden.

Dabei handelt es sich um zwei Tiefziehschienen mit einem okklusalen Aufbau im Oberkiefer als schmaler Steg entlang der Okklusionsflächen von Eckzahn beginnend bis zum letzten Molaren, im Unterkiefer als flache Aufbissebene, auf die der Steg des Oberkiefers idealerweise komplett bei Mundschluss okkludiert. Für die Herstellung dieser zwei Schienen wurde der Patient zunächst zahnmedizinisch gescreent, das bedeutet, ein zahnärztlicher Befund wurde aufgenommen, eventuell geschädigte Zähne wurde konservierend vor Einsatz des Gerätes korrigiert.

Zu diesem Screening gehörte die Feststellung, ob bei dem Freiwilligen eine eingeschränkte Kaufunktion vorhanden war. Hierbei könnten in der späteren Schiene kritische Zähne durchaus ausgeblockt und von der Kaulast befreit werden.

Zum Standardscreening gehörte ebenfalls das Herstellen eines Orthopantomogramms (OPGs), um eine bessere Übersicht vor allem auch über die parodontalen und ossalen Verhältnisse zu bekommen (apikale Geschehen, Knochenabbau bzw. fortgeschrittene Osteolyse) und ob mit den vorhandenen Zähnen genügend Stabilität für die spätere Schiene erzielt werden kann. Die Abdrucknahme erfolgte mit einfachem Alginat. Aufgrund der technisch bedingten Bisssperre durch die spätere Schiene war ein Konstruktionsbiss zwingend erforderlich. Dieser Biss wurde als möglichst leicht geführter Biss mit einer Sperrung von ca. 4 mm am besten aus einem Bissnahmewachs hergestellt. Abdrücke und Bissnahme erfordern eine hohe Präzision.

Im Labor wurden die Modelle mit dem zugehörigen Konstruktionsbiss in einen Mittelwertartikulator eingebracht (nachdem sie dupliziert wurden). Bevor tiefgezogen wird, werden unter sich gehende Stellen mit Wachs oder Kunststoff ausgeblockt. Auf dem duplizierten Modell werden Tiefziehfolien der Stärke 1,0 aus PET-G angefertigt. Dieses Material zeichnet sich nicht nur durch hohe Biokompatibilität, sondern auch durch die Möglichkeit aus, Kunststoff im Nachhinein einzustreuen. Die Folien werden in das Artikulationsmodell eingesetzt und zunächst eine flache Plattform auf die untere Folie mit PMMA (Polymethylmetacrylat), dem üblichen Kunststoff zur Herstellung von Positionierungsschienen aufgebracht. Dies kann entweder im Streuverfahren oder im Anteigverfahren erfolgen. Ein vorheriges Anrauhen der Folie und Benetzen mit Monomer ist sinnvoll. Nach Herstellung der unteren Plattform wird im Oberkiefer der „Kausteg“ aufgestreut. Dieser hat eine Breite von ca. 4 mm und kann im Nachhinein vom Behandler verkleinert werden. Besonders bei nicht perfekter Bissnahme (zu geringer Sperrung) bietet es sich an, die Apparaturen im Mittelwertartikulator herzustellen, da auf diese Weise relativ unproblematisch ein wenig erhöht werden kann. Vorm Aufstreuen des Stegs muss im Unterkiefer die Plattform poliert, und danach isoliert werden.

Der Einsatz am Patienten verläuft in der Regel problemlos. Wichtig ist, darauf hinzuweisen, dass zur Entnahme möglichst im dorsalen Bereich der Schiene angesetzt wird, um das Bruchrisiko der Schiene zu verringern. Der Patient bekommt Instruktionen zur Pflege, wobei die Reinigung mit Zahnpaste und Zahnbürste nach jeder Anwendung reicht. Besonders, da die Schienen nur kurzzeitig (ca. 5 x max. 20 Minuten) getragen werden, entstehen in der Regel keine Verkalkungen und Verschmutzungen. Wichtig ist am Patienten die Überprüfung des gleichmäßigen Aufkommens der Stege auch bei leichten Lateralbewegungen.

Die Schiene wird nur zur Nahrungsaufnahme eingesetzt und direkt danach wieder entnommen und gesäubert. Die erste Kontrolle des Gerätes erfolgt nach 4-6 Wochen. Nach spätestens 8 Wochen wurde die Schiene nicht mehr genutzt. Es ist davon auszugehen, dass nach dieser Zeit sich die Nahrungsaufnahmegeschwindigkeit dauerhaft zum Positiven verändert hat.

**Einschätzung potenzieller Risiken aus kiefernorthopädischer Sicht und individuelle Aufklärung**

Üblicherweise werden Schienen, die in der Zahnmedizin hergestellt werden, nicht explizit zum Essen eingesetzt. Man kann grob folgende Schienen unterscheiden, die in der Zahnmedizin angewandt: Retentionsschienen nach kieferorthopädischer Behandlung/Knirscherschienen, Positionierungs­schienen mit adjustierter Oberfläche und Protrusionsschienen, die eine ganz spezielle Unterkieferposition vorgeben als Apnoe-Schienen.

Der Genusstrainer ähnelt materialtechnisch einer Retentionsschiene. Diese sehr simplen Schienen dienen rein der Erhaltung der Ist-Situation, deswegen auch als Retentionsschienen in der Kieferorthopädie eingesetzt und dem Schutze vor Dysfunktion. Sie haben jedoch keine adjustierte Oberfläche, was der größte Unterschied zwischen den beiden Systemen ist.

Insofern bildet der Genusstrainer eine Innovation. Aus der täglichen Routine ist jedoch bekannt, dass einige Patienten Zahnschienen auch für die Mahlzeiten im Mund belassen. Dies ist in der Regel problemlos. Daher wird die explizite Nutzung einer Schiene zu den Mahlzeiten aus zahnärztlicher und kiefernorthopädischer Sicht nach aktuellem Erkenntnisstand als unbedenklich angesehen.

Risiko: Bedeckung der Zähne durch die Schiene während der Nahrungsaufnahme.

Ausführung: Während der Nahrungsaufnahme kommen tatsächlich kleinste Mengen der Nahrung unter den Bereich der Schiene, weswegen der Patient dazu angehalten wird, keine extrem sauren (Orangensaft trinken) oder färbende Lebensmittel (Rotwein, Kaffee) mit der Schiene aufzunehmen, wobei bei noch keinem einzigen Probanden in irgendeiner Weise Nebenwirkungen zu beobachten waren. Aufgrund des verlängerten Kauablaufs dürfte auch die Speichelsekretion steigen, was wieder einen positiven Aspekt auf Verdauung und Schutz der Zahnhartsubstanzen haben dürfte.

Risiko: Punktuellere Belastung der Zähne und das Parodonts.

Ausführung: Dieser Punkt ist vor allem durch die sorgfältige vorherige Anamnese, radiologischen und intraoralen Befunde abzuklären. Bei Beachtung der oben aufgeführten Maßnahmen können Schäden am Zahnhalteapparat nahezu ausgeschlossen werden.

Risiko: Belastung des Kiefergelenks.

Ausführung: Betreffend die Kiefergelenke ergibt sich natürlich während das Kauvorgangs eine Bisserhöhung von ca. 4 mm. Da die Schiene allerdings nur zur Nahrungsaufnahme eingesetzt wird und die restliche Tageszeit nicht getragen wird und die Behandlungszeit insgesamt nur auf 6-8 Wochen beschränkt bleibt, sind negative Folgen für das Kiefergelenk nahezu ausgeschlossen. Trotzdem bleibt an diesem Punkt die Empfehlung aus kieferorthopädischer Sicht, die Behandlung tatsächlich zeitlich einzuschränken, um sicherzugehen, so dass keinerlei Kiefergelenksproblematik auftreten können. Eine Bisserhöhung im Seitenzahnbereich wird auch in der Prothetik auch nur zu Testphasen regelmäßig eingesetzt, außerdem auch in der Behandlung von CMD-Problematiken im Sinne von okklusalen Splints. Insofern scheint das Risiko der Schädigung in diesem Bereich vernachlässigbar zu sein.

Risiko: Bedenklichkeit des Fremdkörpers bzw. chemische Eigenschaften.
Ausführung: Das von uns verwendete Material PET-G ist ein weit verbreitetes Material, was auch in der Nahrungsmittelindustrie als Verpackungsmaterial häufig genutzt wird. Das Material PMMA (Polymethylmetacrylat) wird zur Herstellung von Prothesen und Spangen genutzt, wobei insbesondere Prothesen während der Nahrungsaufnahme ebenfalls getragen werden, insofern sind die Materialien unbedenklich. Vermieden werden sollte Polycarbonat als Schienenmaterial aufgrund der möglichen Ausschwemmung von Bisphenol während der Aufnahme heißer Speisen und Getränke.

**Mögliche Erklärung des Wirkmechanismus**

Der Effekt der Schiene an sich beruht sicher auf mehreren Prinzipien. Der erhebliche Effekt der Schiene ist, dass der Patient durch Verkleinerung der Kauflächen und auch durch eine gewisse Behinderung durch die Schienen selber, erheblich im Kauen gehemmt wird und dadurch die Nahrungszerkleinerung beim Kauen deutlich zeitintensiver wird. Es sind zum Zerkleinern der Speise je nach deren Beschaffenheit eine deutlich erhöhte Anzahl von Kauabläufen nötig, um den Bolus so zu zerkleinern, so dass er geschluckt werden kann. Insofern verbleibt die Speise länger im Mund, die Nahrungsaufnahme reduziert sich dementsprechend pro Zeiteinheit, das Sättigungsgefühl, das leicht verzögert eintritt, kann sich dementsprechend früher entfalten. Dies bewirkt ein intensiveres Schmecken der Speisen. Dadurch werden die einzelnen Geschmacksstoffe, vor allem die sich langsam entfaltenden deutlicher wahrgenommen und Geschmacksverstärker und billige Fette werden als sehr unangenehm wahrgenommen.

Es besteht weiterhin ein sicher erheblicher psychologischer Effekt durch das Erinnern, die Nahrungsaufnahme zu verlangsamen beim Einsetzen der Schiene, der schon von alleine ein gewisses Ausmaß zum Therapieerfolg beitragen dürfte. Die Mischung aus diesen beiden Effekten (psychologischer und Hemmung bei der Nahrungsaufnahme) soll möglichst eine dauerhafte Umstellung des Essverhaltens bewirken, so dass die Schiene tatsächlich nur 6-8 Wochen genutzt wird.

Die Schiene wird so zum Genusstrainer und es wird vom Innovator die Hypothese aufgestellt, dass ein „Seelenesser“ wieder zum „Instinktesser“ wird, bei dem nachfolgend die Lagerhaltung wieder stimmt.

**Praxis Dr. P. von Seck**

Rheinstraße 31,

65185 Wiesbaden

Tel.: 0611 /300755

**Probandeninformation**

Liebe(r), sehr geehrte(r)………………………………………….,

Sie haben sich eine Zahnschiene, den sogenannten Genusstrainer anpassen lassen, um Gewicht zu verlieren. Wir haben uns zwar schon häufiger über den Effekt, den der Genusstrainer hatte, unterhalten. Jetzt möchten wir die Daten aller Probanden auswerten.

Dafür brauchen wir Ihr Einverständnis.

Wir werden Sie hierfür telefonisch kontaktieren oder einzelne Fragen bei der nächsten Visite in der Praxis stellen. Diese Befragung kann etwa 30 Minuten dauern. Sofern Ihnen schriftliche Unterlagen vorliegen, nutzen wir dies gerne für die Datenerhebung.

Dies ist wichtig, um eine klinische Studie zu erarbeiten, bei der dann der Genusstrainer genau untersucht wird auf seine Wirksamkeit, Sicherheit und seinen Nutzen. Wir haben gesehen, dass das Gewicht über einen längeren Zeitraum bei den einzelnen Teilnehmern abgenommen hat. Aus den genauen Werten und anderer Einflussgrößen, wie Ausgangsgewicht, Alter, Gewichtsverlauf vor und nach Einsatz des Genusstrainers, Medikation, Begleiterkrankungen, Auswirkungen der Gewichtsreduktion auf das tägliche Leben, subjektive Meinung zum Genusstrainer oder sportliche Aktivität.

Durch Ihre Unterschrift auf der Einwilligungserklärung erklären Sie sich damit einverstanden, dass der Studienarzt und seine Mitarbeiter Ihre personenbezogenen Daten zum Zweck der o.g. Auswertung erheben und verarbeiten dürfen. Personenbezogene Daten sind z.B. Ihr Geburtsdatum, Ihr Geschlecht, Daten zu Ihrer physischen und psychischen Gesundheit oder andere persönliche Daten, die während der Auswertung erhoben werden.

Der Studienarzt wird Ihre personenbezogenen Daten für Zwecke der Verwaltung und sowie für Zwecke der Forschung und statistischen Auswertung verwenden.

Der Studienarzt gibt während der Auswertung erhobene studienbezogene Daten an MEDIACC, Medizinisch-academische Beratungsgesellschaft mbH, Gneiststr. 4, 14193 Berlin weiter, welche weitere Untersuchung hierzu durchführt. Die an den vorgenannten Empfänger weitergebenen studienbezogenen Daten enthalten nicht Ihren Namen, Geburtsdatum oder Ihre Adresse. Stattdessen versieht der Studienarzt die Studiendaten mit einer Codenummer (Pseudonymisierung der Daten). Auf den Codeschlüssel, der es erlaubt, die studienbezogenen Daten mit Ihnen in Verbindung zu bringen, haben nur der Studienarzt und seine Mitarbeiter Zugriff. Diese Unterlagen werden, wie auch Ihre übrigen behandlungsrelevanten Unterlagen, für 10 Jahre aufbewahrt. Ihre kodierten Daten werden ausschließlich für die Auswertung dieser Fragestellung und nur von geschulten Mitarbeitern der MEDIACC, die mit diesem Projekt beauftragt sind, vorgenommen.

Sie haben das Recht auf Auskunft über alle beim Studienarzt vorhandenen personenbezogenen Daten über Sie. Sie haben auch Anrecht auf Korrektur eventueller Ungenauigkeiten in Ihren personenbezogenen Daten. Wenn Sie eine Anfrage machen wollen, wenden Sie sich bitte an Ihren Studienarzt. Die Adresse und Telefonnummer des Studienarztes finden Sie im Kopf dieses Formblatts.

Bitte beachten Sie, dass die Ergebnisse der Studie in der medizinischen Fachliteratur veröffentlicht werden können, wobei Ihre Identität jedoch anonym bleibt.

Sie können jederzeit der Weiterverarbeitung Ihrer im Rahmen der o.g. Auswertung erhobenen Daten widersprechen und ihre Löschung bzw. Vernichtung verlangen

**Praxis Dr. P. von Seck**

Rheinstraße 31,

65185 Wiesbaden

Tel.: 0611 /300755

**Einverständniserklärung**

(Version 1.0 vom 19.12.2014)

**Entwicklung eines Genusstrainers zur dauerhaften Gewichtsreduktion bei Übergewicht und Adipositas**

Hiermit erkläre ich, …………………………………………….., geb. am ……………….,

dass ich durch Herrn Dr. von Seck mündlich und schriftlich über das Wesen, die Bedeutung und Tragweite der wissenschaftlichen Auswertung im Rahmen der o.g. Studie informiert wurde und ausreichend Gelegenheit hatte, meine Fragen hierzu in einem Gespräch zu klären.

Ich habe insbesondere die mir vorgelegte Patienteninformation verstanden und eine Ausfertigung derselben und dieser Einwilligungserklärung erhalten.

Mir ist bekannt, dass ich meine Einwilligung jederzeit ohne Angabe von Gründen und ohne nachteilige Folgen insbesondere für meine medizinische Behandlung für mich zurückziehen und einer Weiterverarbeitung meiner Daten jederzeit widersprechen und ihre Löschung verlangen kann.

Ich bin bereit, an der wissenschaftlichen Auswertung teilzunehmen.

Einwilligungserklärung zur Datenverarbeitung

Ich erkläre mich damit einverstanden, dass im Rahmen dieser Studie erhobenen Daten/Angaben über meine Gesundheit verschlüsselt und auf elektronischen Datenträgern aufgezeichnet verarbeitet und die anonymisierten Studienergebnisse veröffentlicht werden.

Auch erkläre ich mich einverstanden, dass meine vorgenannten Daten in pseudonymisierter Form an die MEDIACC, Medizinisch-academische Beratungsgesellschaft mbH, Gneiststr. 4, 14193 Berlin zum Zweck der o.g. Studie übermittelt werden dürfen.

Wiesbaden, den ………………2015 Unterschrift des/der Teilnehmer/in

Hiermit erkläre ich, den/die o.g. Teilnehmer/in am ……... 2015 über Wesen, Bedeutung, Tragweite und Risiken der o.g. Studie mündlich und schriftlich aufgeklärt und ihm/ihr eine Ausfertigung der Information sowie dieser Einwilligungserklärung übergeben zu haben.

Wiesbaden, den ………………2015 Unterschrift des aufklärenden Studienarztes
